# Nuclear protein export pathway in aging therapeutics

**DOI:** 10.18632/aging.102948

**Published:** 2020-03-19

**Authors:** Bulmaro Cisneros, Ian García-Aguirre

**Affiliations:** 1 Department of Genetics and Molecular Biology, Center of Research and Advanced Studies (CINVESTAV-IPN), Mexico City, Mexico

**Keywords:** aging, Hutchinson-Gilford progeria syndrome, progerin, nuclear protein export, CRM1, selinexor

Understanding the molecular basis of aging constitutes one of most exciting and intriguing challenges in biology. Hutchinson-Gilford progeria syndrome is a rare premature aging disorder that recapitulates distinctive features of physiological aging; therefore, it is regarded as a key source of information to understand the mechanisms underlying both pathological and normal aging. HGPS is typically caused by a spontaneous single base-pair substitution (1824 C > T) in the LMNA gene, which activates a cryptic splicing site within exon 11, leading to translation of a mutant lamin A termed progerin [1]. Maturation of lamin A implicates an endoproteolytic cleavage by ZMPSTE24, which removes the last 15 amino acids from pre-lamin A, including the farnesylated C-terminal cysteine. In contrast, progerin remains permanently farnesylated because the mis-splicing event causes a deletion of 50 amino acids within its C-terminus, eliminating then the recognition site for ZMPSTE24. Progerin acts in a dominant gain-of-function manner by aberrantly anchoring to the NE, disturbing thereby a variety of cellular functions [1]. Owing to the harmful effect exerted by progerin on the structure and function of the nucleus, it is expected that nucleocytoplasmic transport of proteins through the nuclear pore complex (NPC), is impaired in the disease. Consistent with this idea, we show for the first time that the CRM1-driven nuclear protein export mechanism is abnormally enhanced in HGPS fibroblasts, due to overexpression of Exportin-1 (XPO1), also known as chromosomal region maintenance 1 (CRM1) (Figure 1). CRM1 is the major transport receptor that exports proteins across the NPC to the cytoplasm, via recognition of the hydrophobic-rich nuclear export signal(s) (NES) present in the cargo molecules [2] (Figure 1).

**Figure 1:**
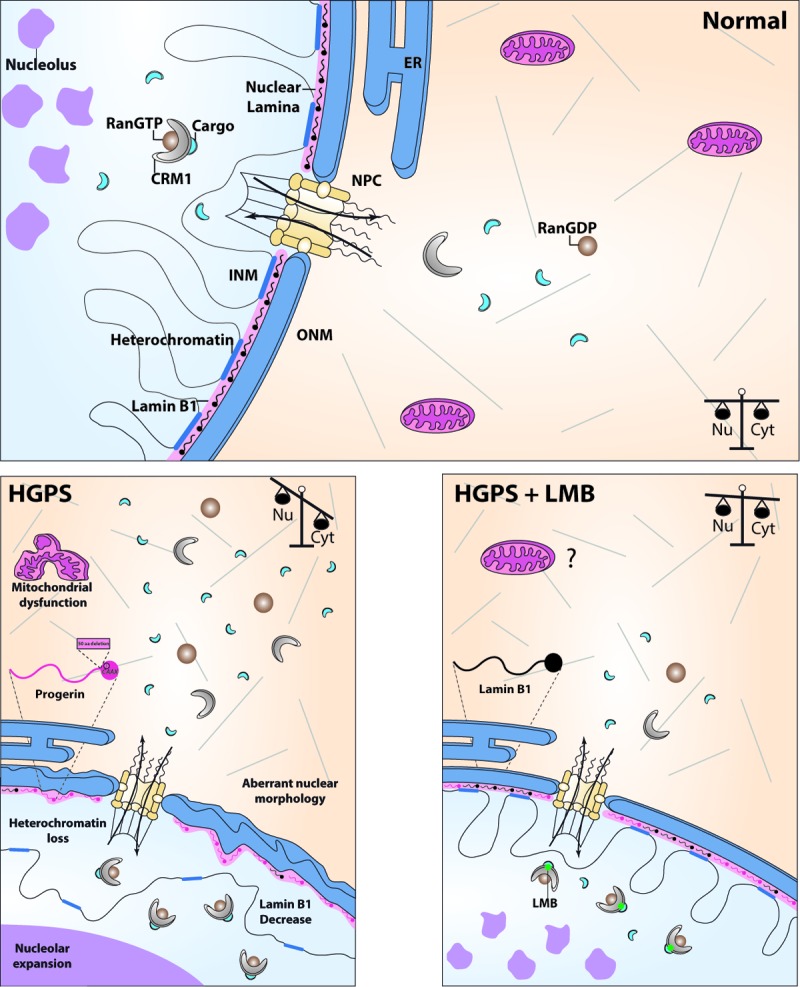
Schematic model showing phenotypic rescue of HGPS cells through pharmacological modulation of CRM1-mediated nuclear export signaling. (**Normal**) CRM1 in complex with Ran-GTP drives the export of proteins from the nucleus (Nu) to the cytoplasm (Cyt) across the nuclear pore complex (NPC), via recognition of a nuclear export signal on the cargo molecules, maintaining thereby a balanced partition of proteins between these cellular compartments. INM, inner nuclear membrane; ONM, outer nuclear membrane; ER endoplasmic reticulum. (**HGPS**) HGPS cells exhibit exacerbated nuclear protein export activity due to progerin-driven CRM1 overexpression, which in turn provokes the appearance of cellular marks of aging, including mitochondrial dysfunction, the loss of heterochromatin, decreased lamin B1 levels, nucleolar expansion and aberrant nuclear morphology. (**HGPS+LMB**) Mitigation of CRM1 activity by treatment of HGPS cells with specific CRM1 inhibitor (LMB) alleviates all aforementioned aging marks, by restoring proper nuclear-cytoplasmic distribution of proteins.

Enhanced nuclear protein export can impact protein homeostasis by altering nucleocytoplasmic partitioning of critical proteins (transcription factors, enzymes, and structural proteins); thus, we hypothesized that perturbation of this central process might be a main contributor to HGPS. As proof-of-concept, we evaluated whether attenuation of nuclear export activity, using a specific inhibitor of CRM1 termed leptomycin B (LMB), exerts a therapeutic effect on the HGPS cellular phenotype. Consistent with this paradigm, treatment of primary HGPS fibroblasts with LMB alleviated virtually all aging marks of HGPS cells, including aberrant nuclear morphology, nucleolar expansion, cellular senescence, loss of peripheral heterochromatin, and lamin B1 downregulation [3] (Figure 1). Consistently, ectopic overexpression of CRM1 was sufficient to recapitulate aging hallmarks in normal fibroblasts (cellular senescence, depleted lamin B1 levels, and the loss of peripheral chromatin) [3]. As the loss of proteostasis is a feature of physiological aging, we hypothesized that CRM1 nuclear protein export pathway could be altered in normal aging too. CRM1 augmented levels were found in human fibroblast from healthy aged donors [3]. Thus, enhanced CRM1 activity is a common mechanism of normal and premature aging

Interestingly, various important cellular processes found as altered in HGPS cells are modulated by CRM1-target proteins: (a) NAD-dependent deacetylase sirtuin 2 (SIRT2) is involved in heterochromatin organization; (b) B23 is a central protein for nucleoli function; (c) dystrophin Dp71, β-dystrobrevin and β-dystroglycan are implicated in nuclear envelope function; and (d) p53 critically modulates cellular senescence. Thus, administration of CRM1 inhibitors will preserve the nuclear fraction of these and many other NES-contained proteins, improving thereby global cellular physiology. Future application of omics technologies is required to fully delineate metabolic, and molecular pathways underlying the therapeutic properties of CRM1 inhibitors on aging.

It is worth to note that patients with HGPS develop cardiovascular disease, characterized by atherosclerosis and cardiac electrophysiological defects, which ultimately lead them to early death due to myocardial infarction or stroke [4]. Interestingly, abnormal increase in nuclear protein export is an early event in the development of cardiac hypertrophy [5], because histone deacetylase 5 (HDAC5) is shuttled out of the cardiomyocyte nucleus in a CRM1-dependent manner in response to hypertrophy signaling, which consequently impedes its action as a repressor of pro-hypertrophic genes [6]. Remarkably, treatment of cardiomyocytes with a CRM1 inhibitor (selinexor) repressed pathological gene expression and linked hypertrophy [6]. Thus, cardiac hypertrophy associated to both HGPS and normal aging would be prevented/delayed or even reversed by pharmacological attenuation of CRM1 activity.

In summary, pharmacological modulation of the CRM1-mediated nuclear export pathway clearly provides a viable and promising therapy against HGPS and other aging-related diseases. The development of synthetic selective inhibitors of CRM1 with pharmacological properties superior to LMB (i.e. selinexor/KPT-330), which have shown to be well-tolerated in cancer clinical trials [7], would facilitate preclinical evaluation of this therapy in HGPS mice models.

## References

[1] Eriksson M, et al. Nature. 2003; 423:293–98. 10.1038/nature01629 12714972 PMC10540076

[2] Ishizawa J, et al. Pharmacol Ther. 2015; 153:25–35. 10.1016/j.pharmthera.2015.06.001 26048327 PMC4526315

[3] García-Aguirre I, et al. Aging Cell. 2019; 18:e13002. 10.1111/acel.13002 31305018 PMC6718587

[4] Hamczyk MR, et al. Annu Rev Physiol. 2018; 80:27–48. 10.1146/annurev-physiol-021317-121454 28934587

[5] Chahine MN, et al. Cardiovasc Res. 2015; 105:31–43. 10.1093/cvr/cvu218 25341891 PMC4277256

[6] Harrison BC, et al. Mol Cell Biol. 2004; 24:10636–49. 10.1128/MCB.24.24.10636-10649.2004 15572669 PMC533968

[7] Mahipal A, Malafa M. Pharmacol Ther. 2016; 164:135–43. 10.1016/j.pharmthera.2016.03.020 27113410

